# Supplementary material for: Calcitriol in the Presence of Conditioned Media from Metastatic Breast Cancer Cells Enhances Ex Vivo Polarization of M2 Alternative Murine Bone Marrow-Derived Macrophages
Source: Cancers (Basel). 2020 Nov 23;12(11):3485. doi: 10.3390/cancers12113485 (PMC7700498; doi:10.3390/cancers12113485)
Supplement: Supplementary file 1 [file cancers-12-03485-s001.pdf]

Supplementary Materials: Calcitriol in the Presence of Conditioned Media from Metastatic Breast Cancer Cells Enhances Ex Vivo Polarization of M2 Alternative Murine Bone Marrow-Derived Macrophages

Artur Anisiewicz, Natalia Łabędź, Izabela Krauze and Joanna Wietrzyk

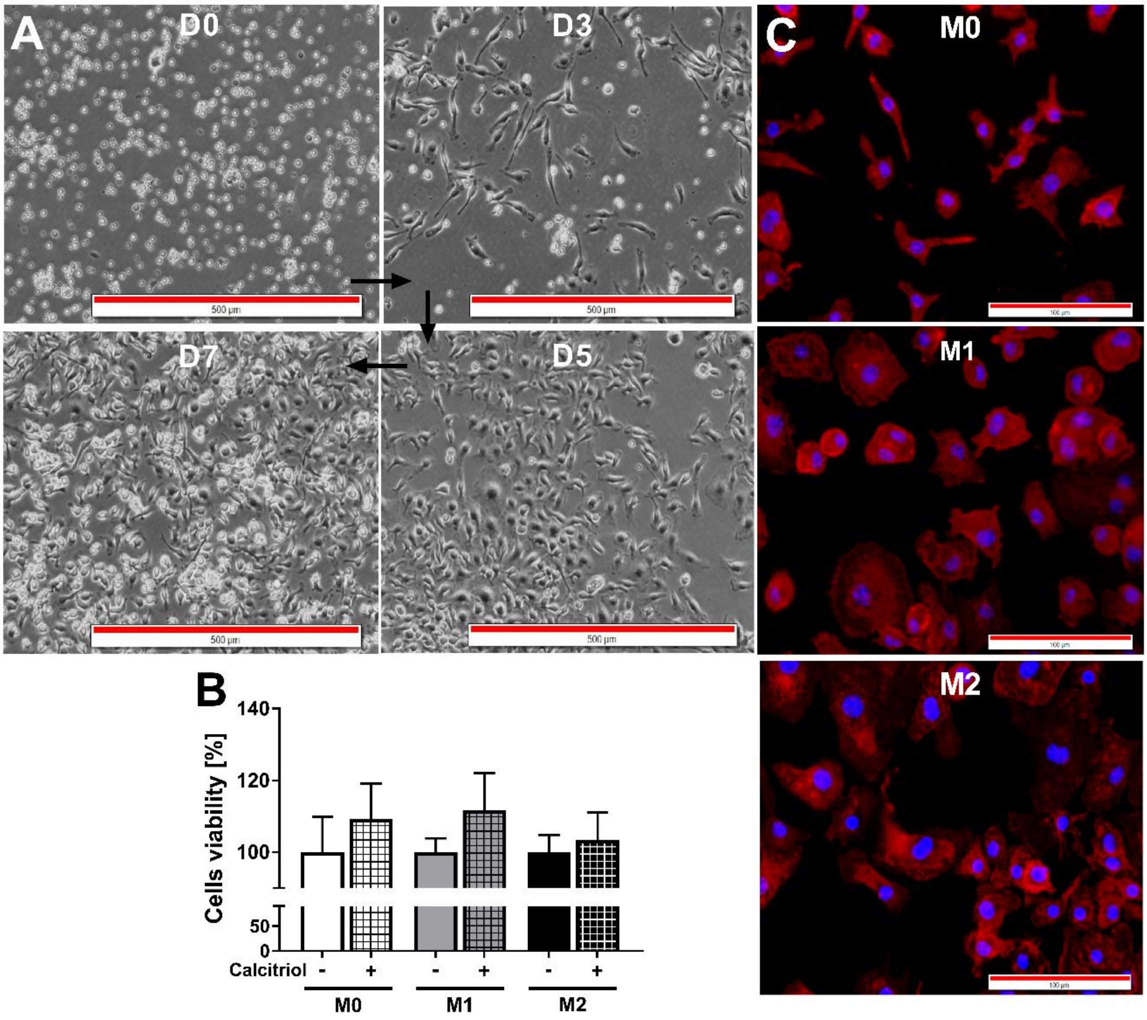

Figure S1. Differentiation and polarization of BMDMs and the effect of calcitriol on their proliferation. Representative images of (**A**) differentiating BMDMs on selected days (D0, D3, D5, D7) under a light microscope (magnification: 10 times, scale bars = 500µm) and (**C**) BMDMs polarized to class M0, M1, and M2 (magnification: 40 times, scale bars = 100µm) visualized with DAPI (cell nuclei, blue) and Phalloidin (actin, red) under a fluorescence microscope. (**B**) The effect of calcitriol on the proliferation of polarized BMDMs estimated in the SRB test. Absorbance results from the BMDMs of individual classes (M0, M1, M2) treated with calcitriol were referred to individual BMDMs polarized without calcitriol (control, 100%). Data presentation: (**B**) mean with standard deviation. Number of independent repetitions = 4 (BMDMs cultures generated from four mice).

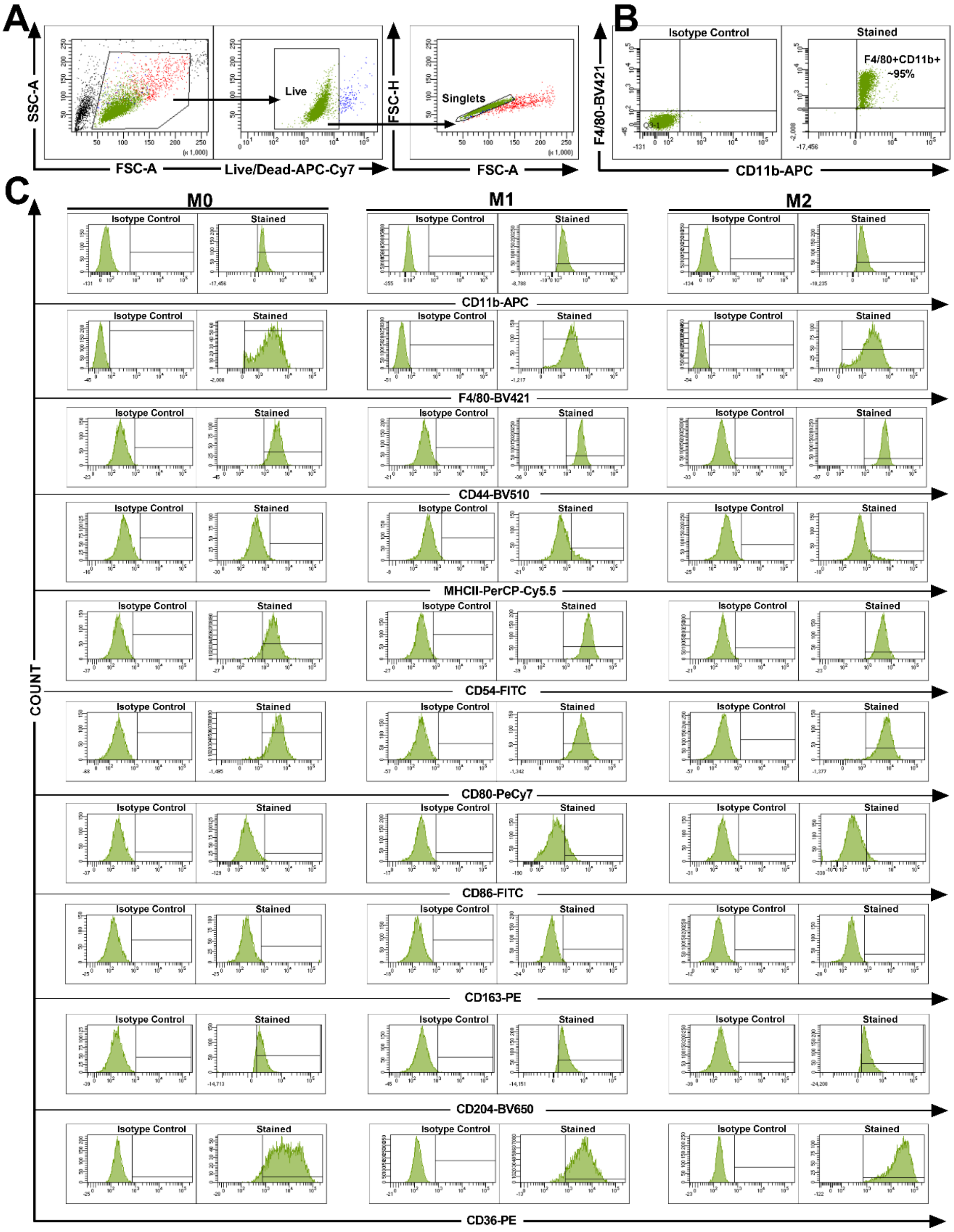

Figure S2. The effect of calcitriol on the expression of BMDMs surface markers of individual classes by FACS analysis. (**A**) The gating strategy, (**B**) purity of the BMDMs cultures and (**C**) images of representative histograms for each BMDMs class. (**A**) The population of analyzed cells was isolated according to the parameters FSC-A and SSC-A, then living cells were separated from dead cells using the Live / Dead Stain Kit. Singlets (FSC-H: FSC-A) were analyzed for the fluorescence intensity of the fluorochromes conjugated with individual antibodies. (**B**) The purity of macrophage cultures determined by CD11b+ and F4/80+ expression was about 95%. (**C**) Histogram gates were set for cells incubated with the appropriate isotype controls, then % of positive cells and fluorescence intensity were analyzed. Number of independent repetitions = 3 (BMDMs cultures generated from three mice).

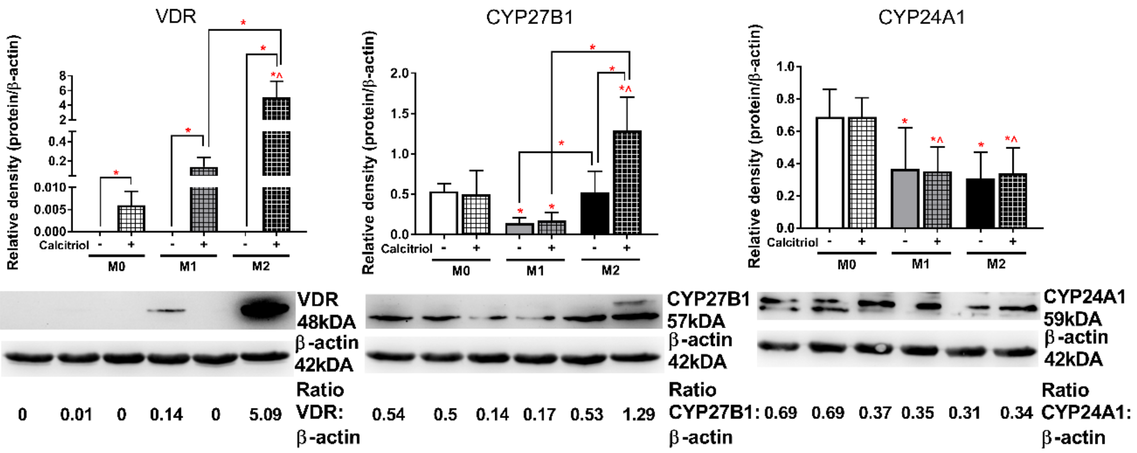

Figure S3. The effect of calcitriol on the protein expression of BMDMs by Western-Blot analysis. Expression of VDR, CYP27B1 and CYP24A1 in macrophage culture lysates and representative blot images. The analysis was carried out in ImageJ software, the results of densitometry for the tested proteins were normalized to the reference protein β-actin. Ratio of tested protein:β-actin is included for each band of each blot. Statistical analysis: Sidak's or Dunn's multiple comparisons test. * $p < 0.05$ as compared to M0, ^ $p < 0.05$ as compared to M0 + cal or as indicated.

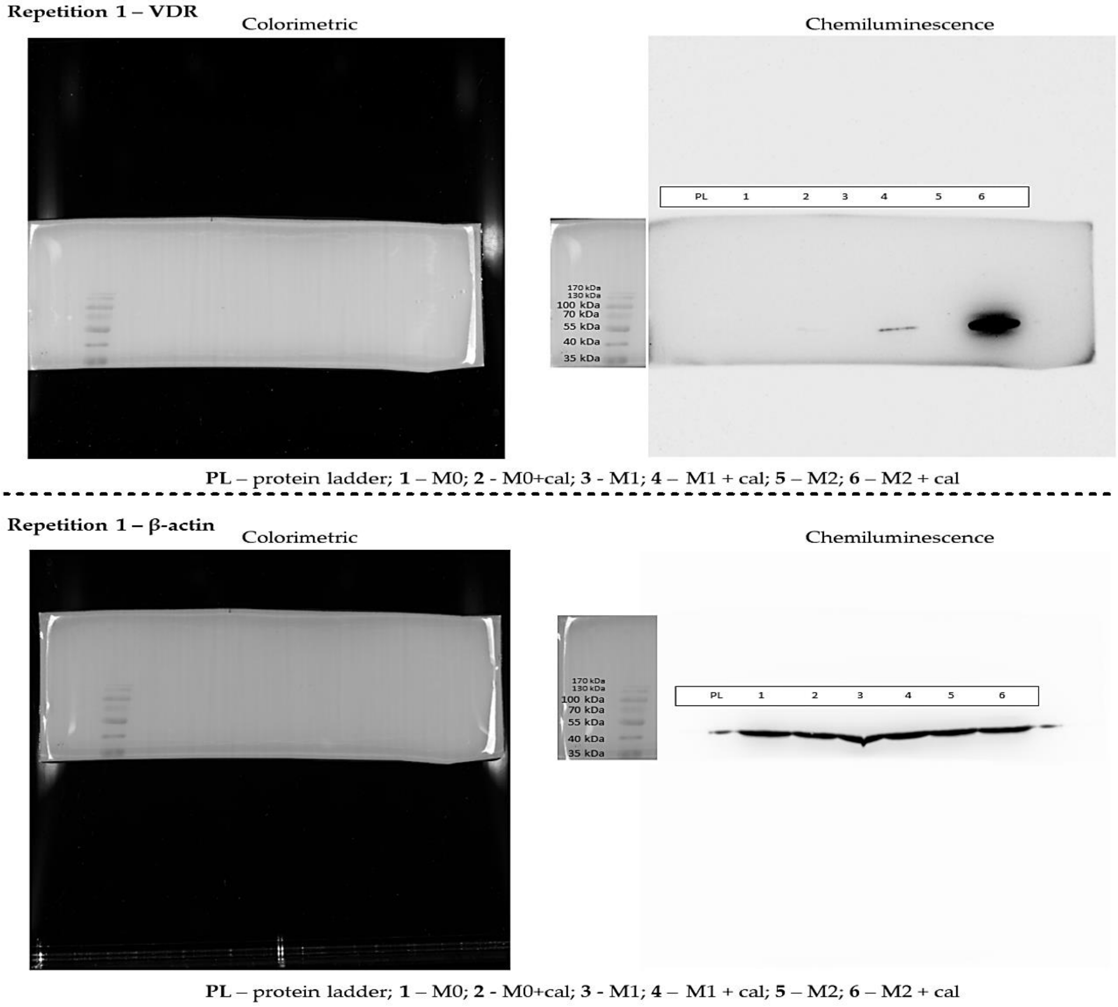

Figure S4. Uncropped Western Blot images: VDR and corresponding β-actin, repetition 1.

Repetition 2 – VDR

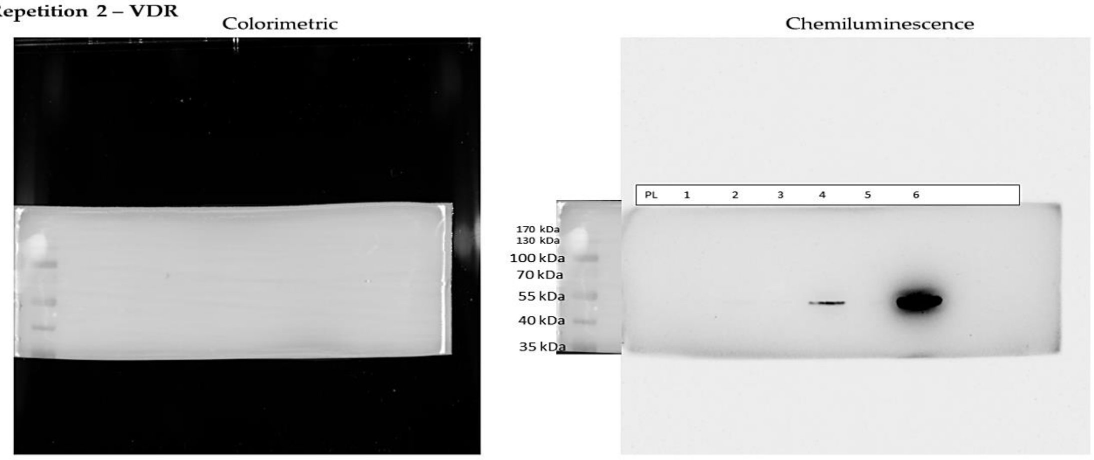

PL – protein ladder; **1** – M0; **2** - M0+cal; **3** - M1; **4** – M1 + cal; **5** – M2; **6** – M2 + cal

Repetition 2 – β-actin

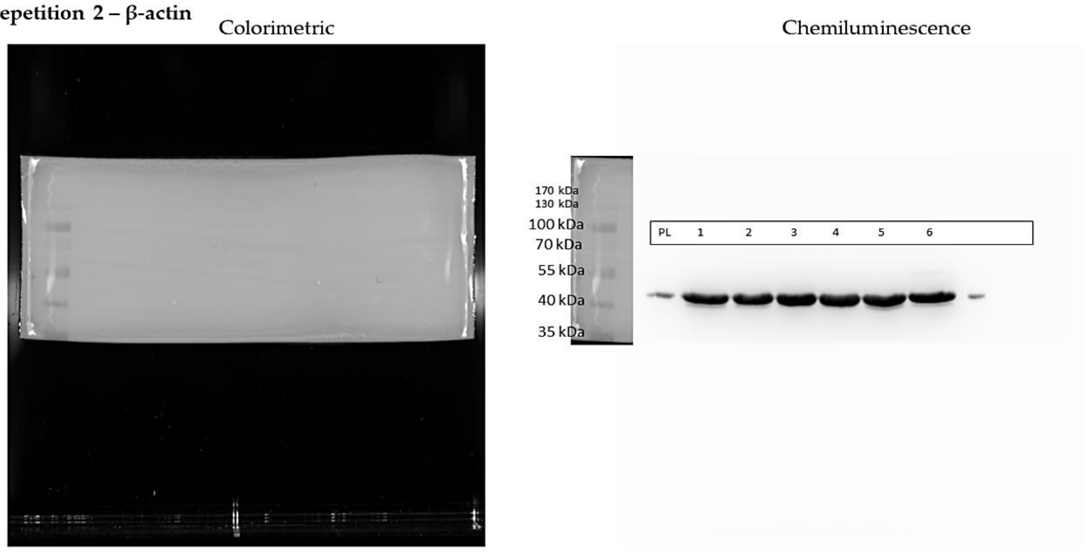

PL – protein ladder; **1** – M0; **2** - M0+cal; **3** - M1; **4** – M1 + cal; **5** – M2; **6** – M2 + cal

Figure S5. Uncropped Western Blot images: VDR and corresponding β-actin, repetition 2.

Repetition 3 – VDR

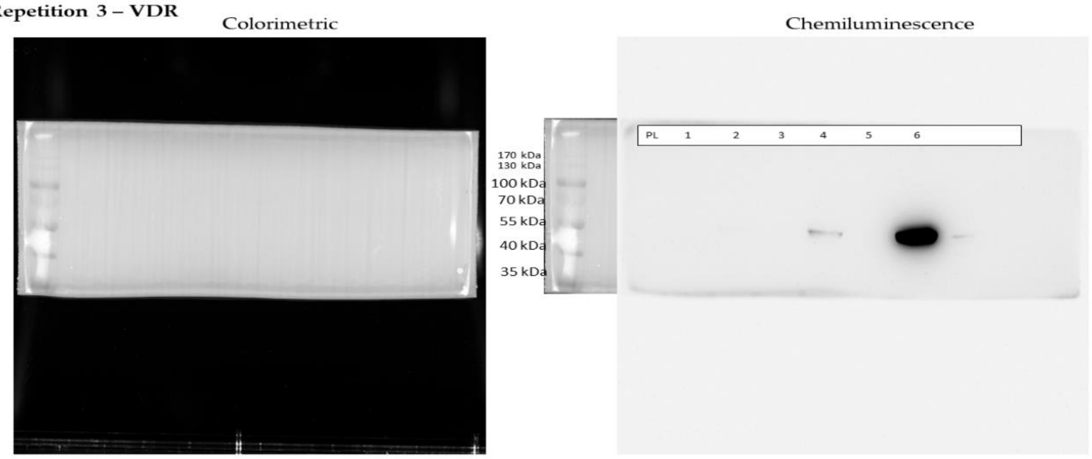

PL – protein ladder; **1** – M0; **2** - M0+cal; **3** - M1; **4** – M1 + cal; **5** – M2; **6** – M2 + cal

Repetition 3 – β-actin

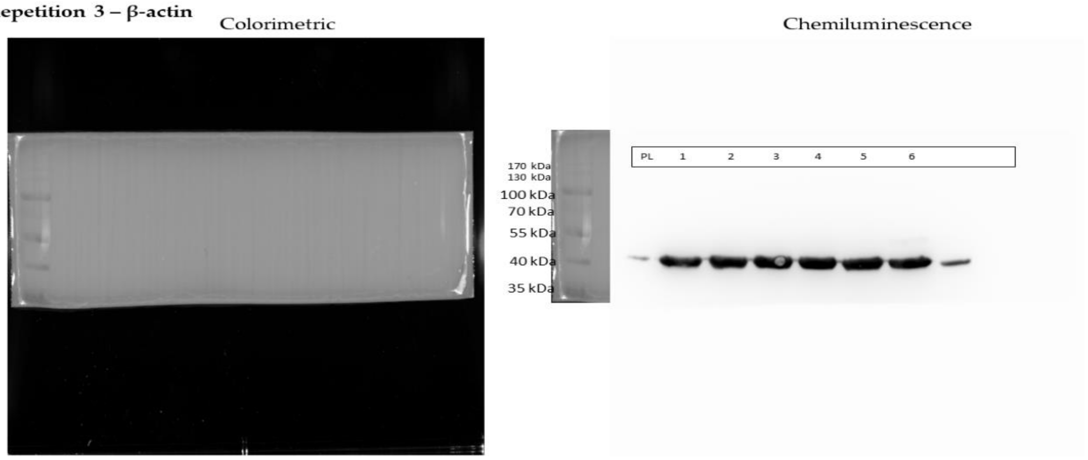

PL – protein ladder; **1** – M0; **2** - M0+cal; **3** - M1; **4** – M1 + cal; **5** – M2; **6** – M2 + cal

Figure S6. Uncropped Western Blot images: VDR and corresponding β-actin, repetition 3.

Repetition 4 – VDR

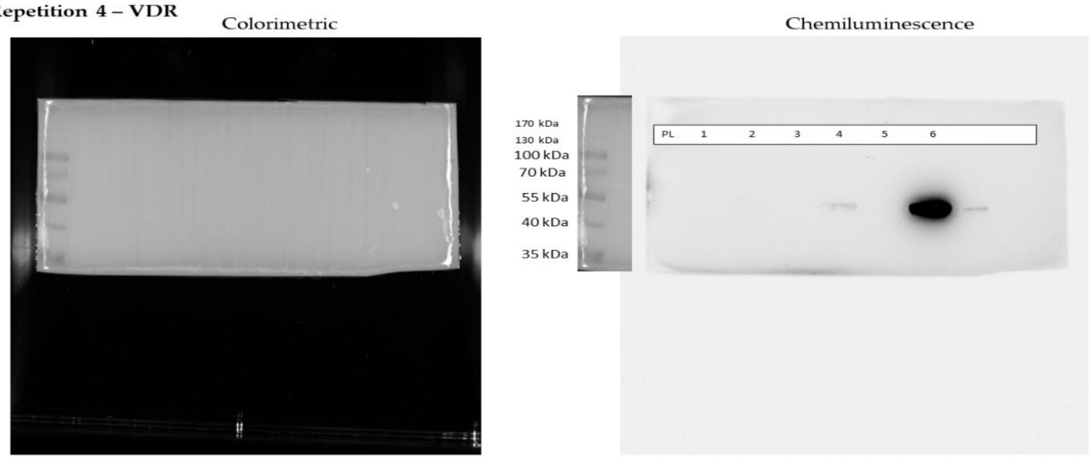

PL – protein ladder; **1** – M0; **2** - M0+cal; **3** - M1; **4** – M1 + cal; **5** – M2; **6** – M2 + cal

Repetition 4 – β-actin

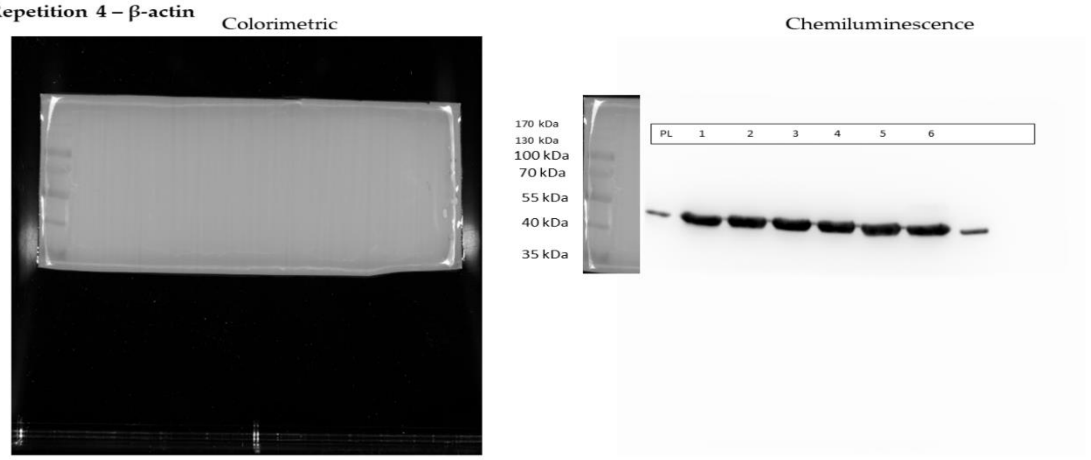

PL – protein ladder; **1** – M0; **2** - M0+cal; **3** - M1; **4** – M1 + cal; **5** – M2; **6** – M2 + cal

Figure S7. Uncropped Western Blot images: VDR and corresponding β-actin, repetition 4.

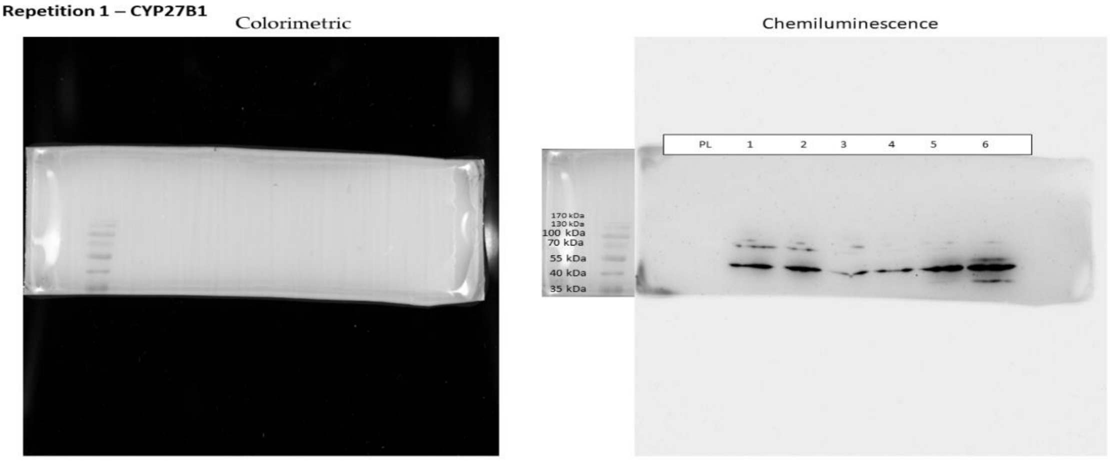

PL – protein ladder; **1** – M0; **2** - M0+cal; **3** - M1; **4** – M1 + cal; **5** – M2; **6** – M2 + cal

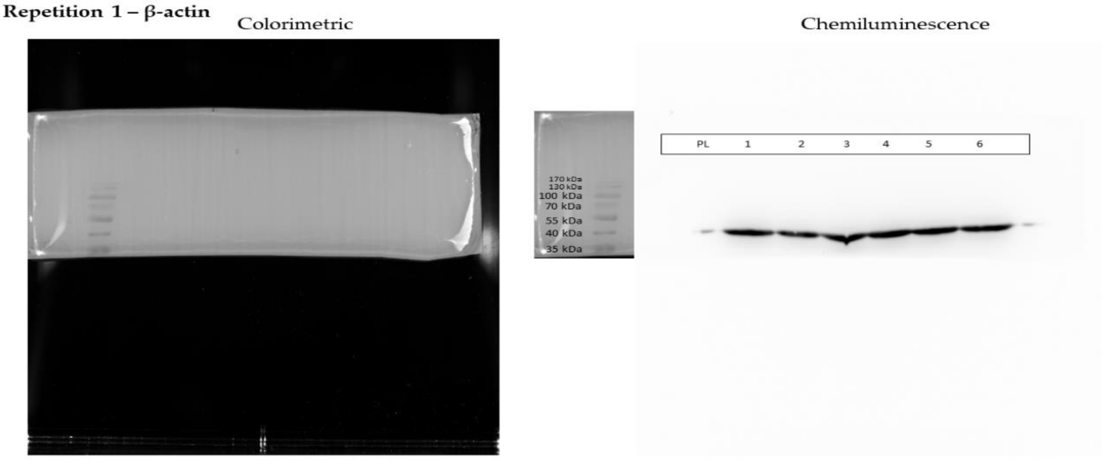

PL – protein ladder; **1** – M0; **2** - M0+cal; **3** - M1; **4** – M1 + cal; **5** – M2; **6** – M2 + cal

Figure 8. SUncropped Western Blot images: CYP27B1 and corresponding β-actin, repetition 1.

Repetition 2 – CYP27B1

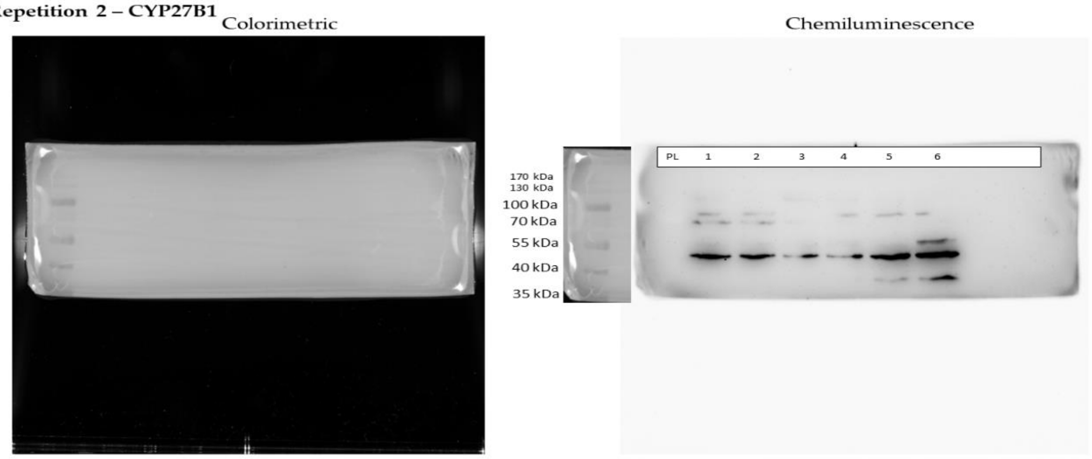

PL – protein ladder; **1** – M0; **2** - M0+cal; **3** - M1; **4** – M1 + cal; **5** – M2; **6** – M2 + cal

Repetition 2 – β-actin

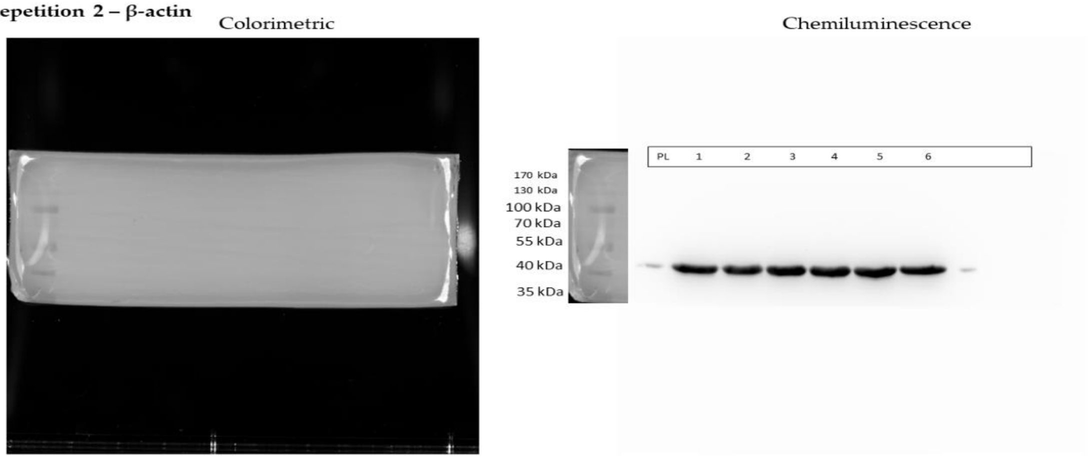

PL – protein ladder; **1** – M0; **2** - M0+cal; **3** - M1; **4** – M1 + cal; **5** – M2; **6** – M2 + cal

Figure S9. Uncropped Western Blot images: CYP27B1 and corresponding β-actin, repetition 2.

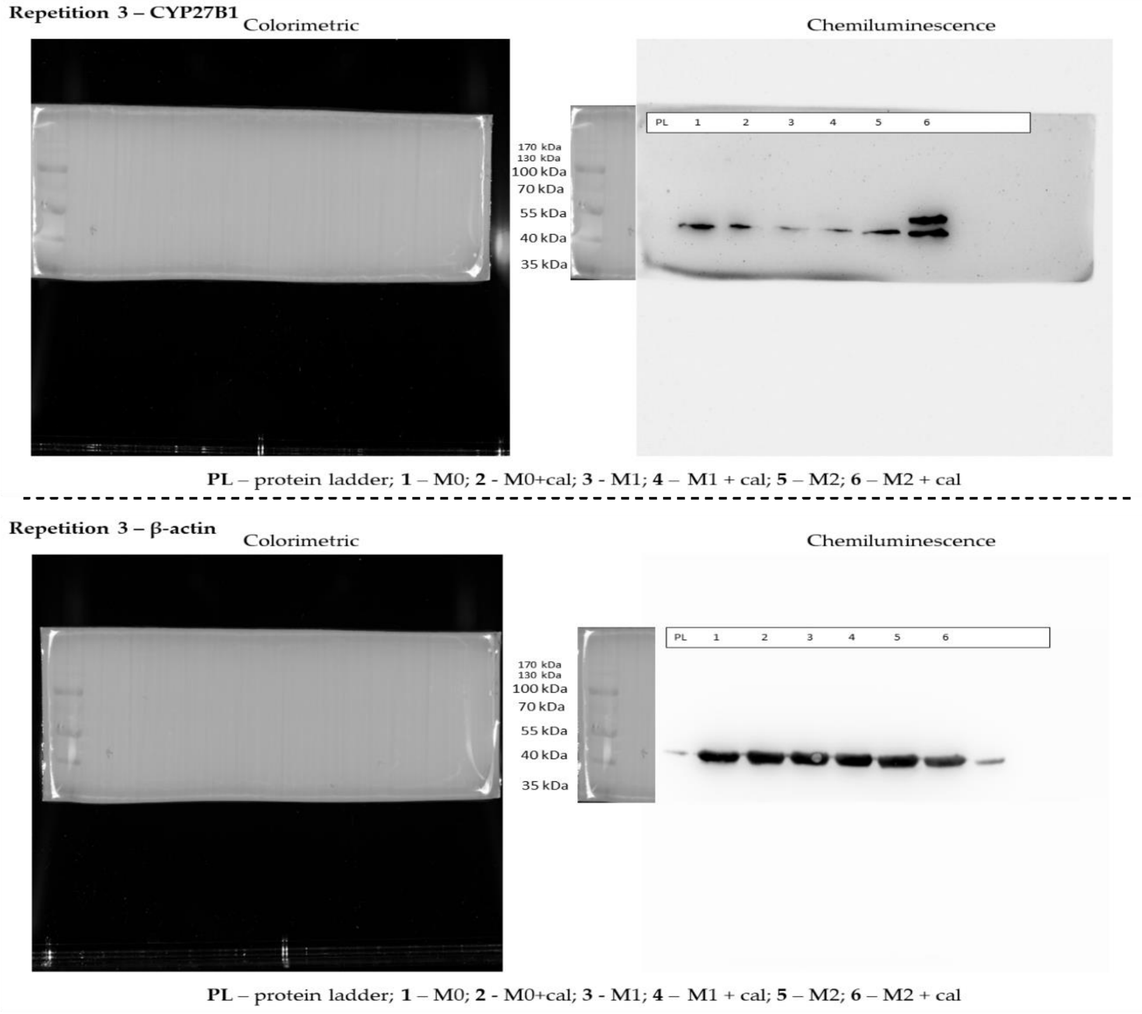

Figure S10. Uncropped Western Blot images: CYP27B1 and corresponding β-actin, repetition 3.

Repetition 4 – CYP27B1

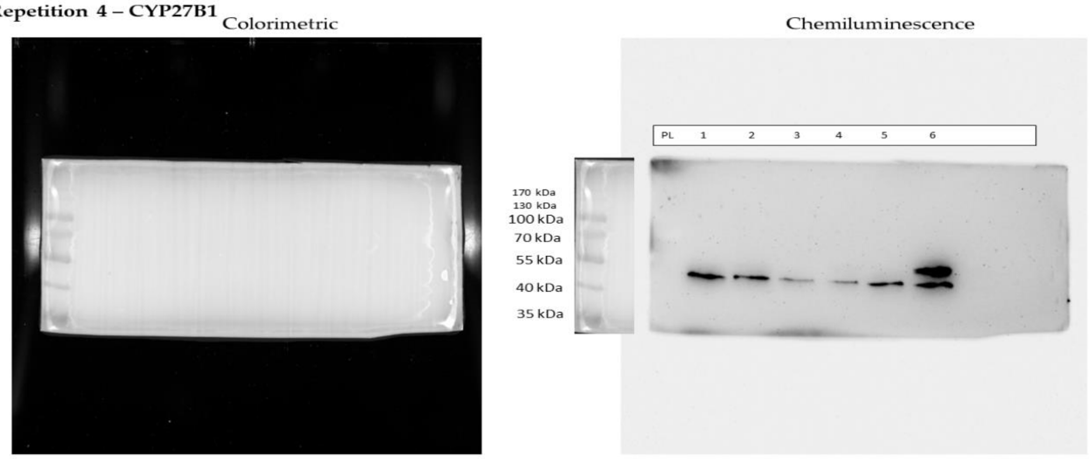

PL – protein ladder; **1** – M0; **2** - M0+cal; **3** - M1; **4** – M1 + cal; **5** – M2; **6** – M2 + cal

Repetition 4 – β-actin

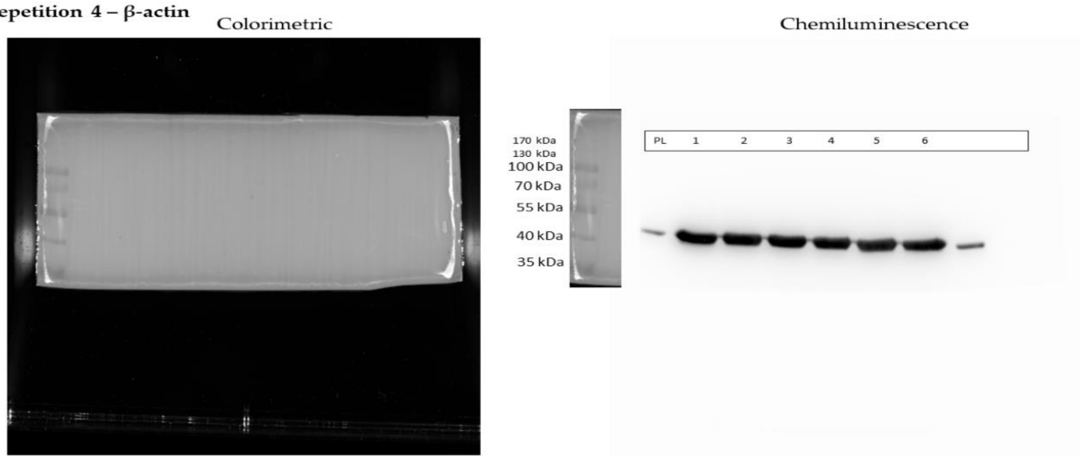

PL – protein ladder; **1** – M0; **2** - M0+cal; **3** - M1; **4** – M1 + cal; **5** – M2; **6** – M2 + cal

Figure S11. Uncropped Western Blot images: CYP27B1 and corresponding β-actin, repetition 4.

Repetition 1 – CYP24A1

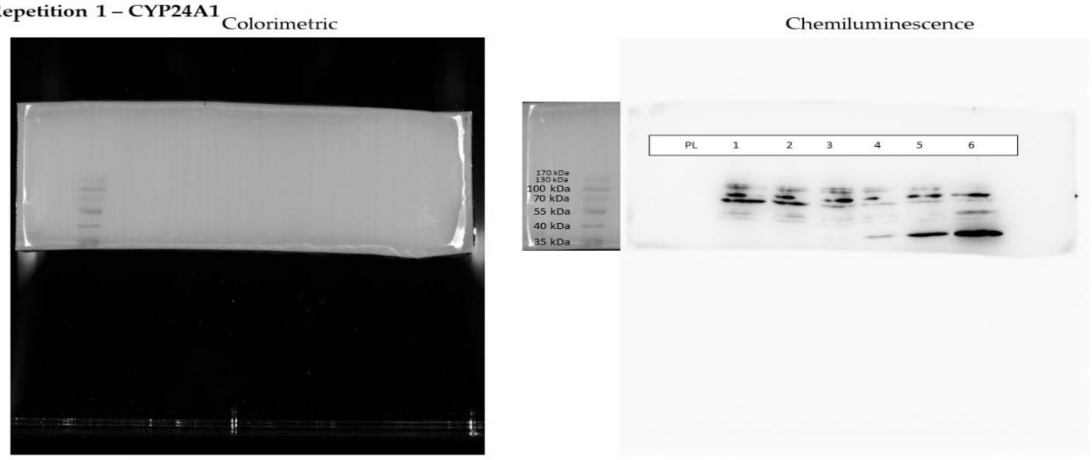

PL – protein ladder; **1** – M0; **2** - M0+cal; **3** - M1; **4** – M1 + cal; **5** – M2; **6** – M2 + cal

Repetition 1 – β-actin

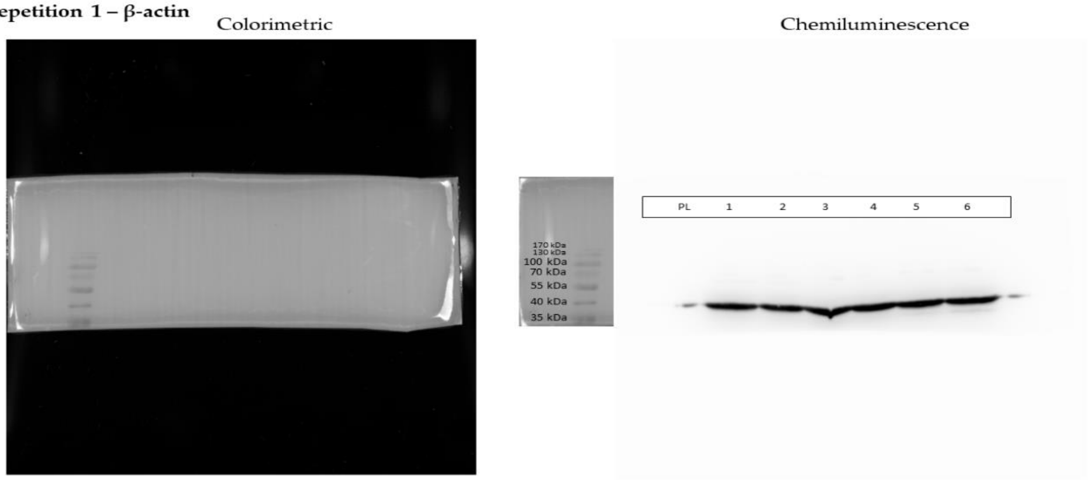

PL – protein ladder; **1** – M0; **2** - M0+cal; **3** - M1; **4** – M1 + cal; **5** – M2; **6** – M2 + cal

Figure S12. Uncropped Western Blot images: CYP24A1 and corresponding β-actin, repetition 1.

Repetition 2 – CYP24A1

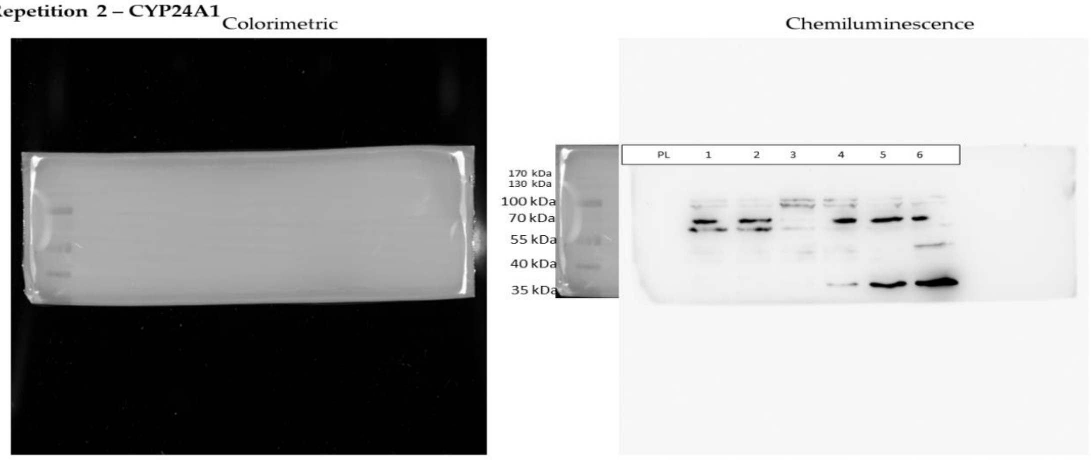

PL – protein ladder; **1** – M0; **2** - M0+cal; **3** - M1; **4** – M1 + cal; **5** – M2; **6** – M2 + cal

Repetition 2 – β-actin

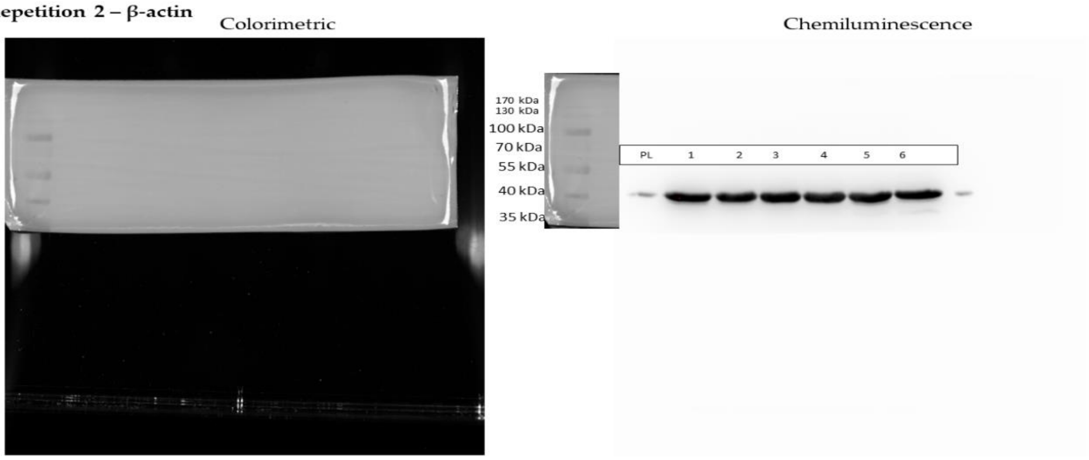

PL – protein ladder; **1** – M0; **2** - M0+cal; **3** - M1; **4** – M1 + cal; **5** – M2; **6** – M2 + cal

Figure S13. Uncropped Western Blot images: CYP24A1 and corresponding β-actin, repetition 2.

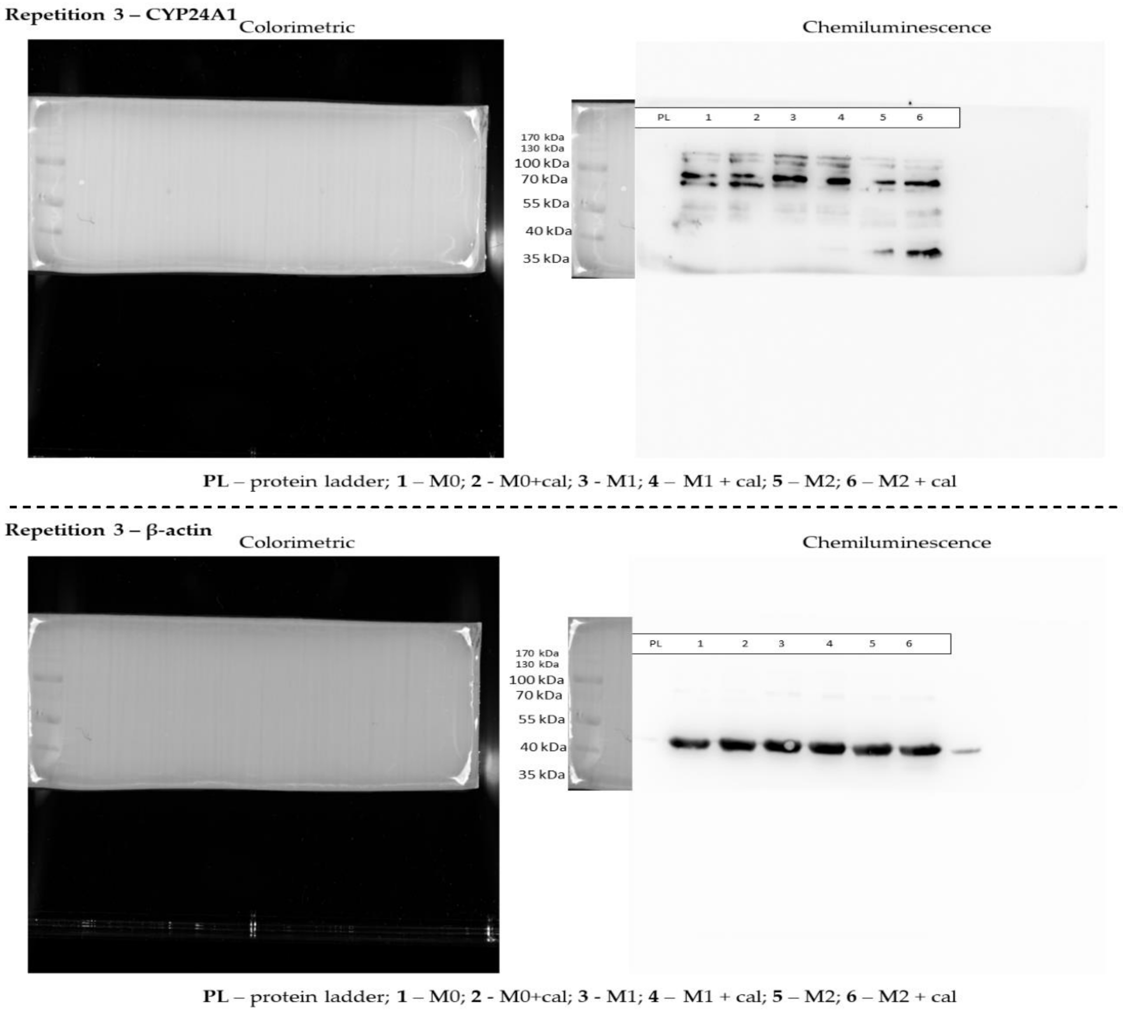

Figure S14. Uncropped Western Blot images: CYP24A1 and corresponding β-actin, repetition 3.

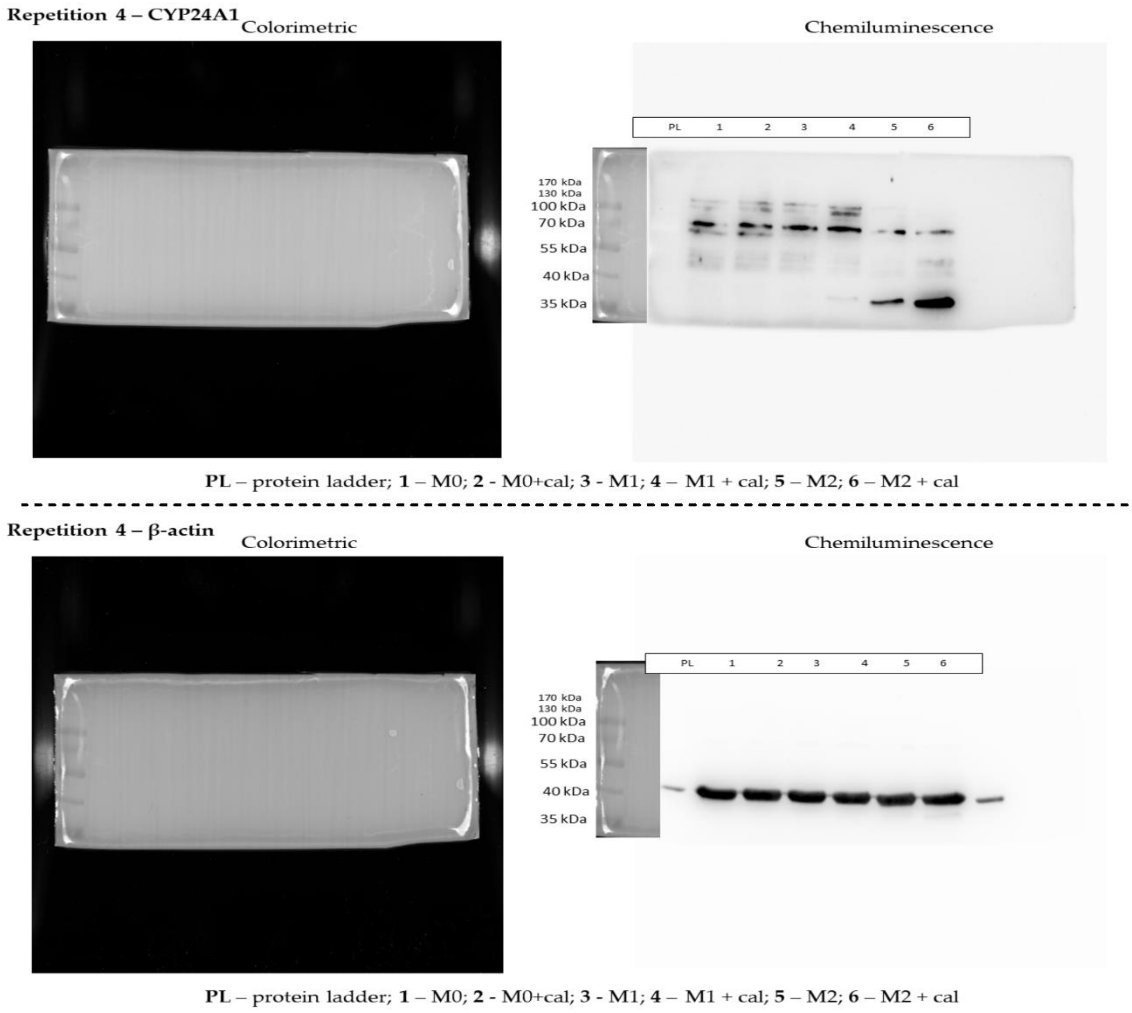

Figure S15. Uncropped Western Blot images: CYP24A1 and corresponding β-actin, repetition 4.

Table S1. Production of cytokines and chemokines to cell culture supernatants by BMDMs polarized in the presence or absence of calcitriol.

Protein	M0	M0 + cal	M1	M1 + cal	M2	M2 + cal
G-CSF	1.36% ± 0.24	0.81% ± 0.50	0.04% ± 0.08	1.45% ± 0.53	3.10%*# ± 1.14	3.61%*^# ± 0.47
GM-CSF	0.22% ± 0.24	0.04% ± 0.08	0.00% ± 0.00	0.00% ± 0.00	4.88%*# ± 1.11	3.67%*^# ± 0.23
IFN-γ	0.00% ± 0.00	0.00% ± 0.00	0.02% ± 0.05	1.00%*^& ± 0.41	0.00% ± 0.00	0.00%# ± 0.00
IL-1RA	20.50% ± 2.08	13.34% ± 5.34	79.44%* ± 18.23	104.76%*^& ± 6.31	55.42%*# ± 10.62	49.91%*^# ± 13.07
IL-10	0.00% ± 0.00	0.00% ± 0.00	0.00% ± 0.00	0.00% ± 16.05	1.32%*# ± 0.51	1.09%*^# ± 1.36
IL-27	0.00% ± 0.00	0.00% ± 0.00	0.00% ± 0.00	0.00% ± 0.00	4.56%*# ± 1.82	6.31%*^# ± 2.29
CXCL10	71.66% ± 11.99	45.75%* ± 9.43	100.04%* ± 8.77	90.25% ± 6.50	121.41%* ± 21.16	123.68%*^ ± 8.23
CXCL11	1.92% ± 0.47	1.25% ± 0.28	1.08%* ± 1.04	2.41%^& ± 0.48	0.00%*# ± 0.00	0.00%*^# ± 0.00
CXCL1	69.48%	68.10%	16.13%*	20.79%*^	124.39%*#	143.05%*^#&

	± 4.02	± 8.14	± 6.32	± 4.81	± 14.06	± 16.28
CCL12	0.00% ± 0.00	0.00% ± 0.00	0.00% ± 0.00	0.00% ± 0.00	5.44%*# ± 8.05	3.76%*^# ± 2.84
CXCL9	2.00% ± 0.40	1.07% ± 0.45	13.80%* ± 5.22	12.27%*^ ± 4.34	6.89%*# ± 0.91	4.01% ± 2.70
CCL3	101.63% ± 11.21	88.85% ± 15.65	1.82%* ± 0.98	5.72%*^ ± 1.90	100.13%# ± 10.42	77.59%*#& ± 16.36
CCL4	79.07% ± 11.44	72.03% ± 16.89	0.49%* ± 0.39	3.41%*^ ± 0.86	69.20%# ± 9.51	66.37%# ± 14.48
CXCL2	110.57% ± 11.62	104.17% ± 12.07	49.29%* ± 7.50	52.74%*^ ± 4.53	83.07%*# ± 12.06	67.31%*^ ± 16.51
CCL5	136.22% ± 15.65	126.49% ± 13.37	120.81% ± 15.07	105.06%*^ ± 6.69	121.55% ± 13.29	110.80%* ± 4.31
CXCL12	6.45% ± 1.48	4.79% ± 2.16	4.78% ± 0.65	5.40% ± 2.80	3.46%* ± 0.84	3.44%* ± 0.94
TIMP-1	2.11% ± 0.54	1.55% ± 0.23	1.52% ± 1.25	2.97% ± 0.77	13.64%*# ± 5.24	17.58%*^# ± 5.63
TNF-α	10.82% ± 4.06	13.76% ± 3.88	109.60%* ± 14.78	115.36%*^ ± 17.10	46.21%*# ± 4.76	48.80%*^# ± 7.68

The expression value of the tested proteins was presented as a percentage, in relation to the positive control placed on the membrane by the producer (100% value). Data presentation: mean. Number of independent repetitions = 4 (BMDMs cultures generated from four mice). Statistical analysis: Sidak's or Dunn's multiple comparisons test. * $p < 0.05$ as compared to M0, ^ $p < 0.05$ as compared to M0+cal, # $p < 0.05$ as compared between M1-M2 or M1 + cal-M2 + cal, & $p < 0.05$ as compared between M1-M1 + cal or M2-M2 + cal.

Publisher's Note: MDPI stays neutral with regard to jurisdictional claims in published maps and institutional affiliations

© 2020 by the authors. Licensee MDPI, Basel, Switzerland. This article is an open access article distributed under the terms and conditions of the Creative Commons Attribution (CC BY) license (http://creativecommons.org/licenses/by/4.0/).
